# Apocrine carcinoma in the parotid gland and in the submandibular region

**DOI:** 10.1016/S1808-8694(15)31314-8

**Published:** 2015-10-20

**Authors:** Jairo S. Francisco, Silvia E.N. Alfaro, Daniela C. C.M. Oliveira, Sebastião Tonon, Eliane P. Dias

**Affiliations:** 1 Master studies in Buccodental Pathology under course - UFF; 2 Master in Buccodental Pathology; 3 Specialization in Otorhinolaryngology under course – University Hospital Antônio Pedro/UFF; 4 Joint Professor, Specialization in Otorhinolaryngology – University Hospital, Antônio Pedro/UFF; 5 Coordinator of Master Program in Buccodental Pathology, UFF

**Keywords:** salivary glands diseases, skin neoplasms, apocrine glands pathology

## Abstract

The objectives of this paper are to report a case of apocrine carcinoma and the discussion of aspects related to its diagnosis, treatment, and prognosis. Carcinomas with apocrine differentiation not related to extramammary Paget's disease, ductal breast carcinoma, Moll's glands adenocarcinoma and ceruminous glands carcinoma are very uncommon tumors. We report a case of a 51-year-old black woman who developed apocrine carcinoma lesions in the head and neck region. Two lesions involved her left parotid gland (first tumor and local recurrence), and other involved her submandibular skin. The microscopic aspects were as follows: infiltrative glandular epithelial neoplasm with moderate cellular and nuclear pleomorphism; neoplasic cells with polygonal or circular shape, large nuclei and eosinophilic and granular cytoplasm. The apical decapitation secretion was viewed in a large number of intra-cystic tumor cells. Moreover, we found areas with comedo-necrosis or PAS positive staining (with or without diastase). Based on cutaneous apocrine carcinoma compatibility of the microscopic aspects, we concluded that the tumor in the submandibular skin was probably the primary neoplasm. The patient was treated by surgical excisions, and no evidence of recurrent or metastatic disease has been seen after a follow-up period of 12 months.

## INTRODUCTION

Apocrine secretion glands in human beings are represented by apocrine sweat glands located in the armpits, anogenital region and breast aureole, in addition to Moll's glands in the palpebra and wax ear glands; there are very rare reports of onset of apocrine differentiated carcinomas in any region apart from those^1^. According to Hayes et al.^2^ and Paties et al.^3^, forms of carcinoma with apocrine differentiation are normally related with extramammary Paget disease, breast ductal carcinoma, adenocarcinoma of Moll's glands, and ceruminal carcinoma. The onset of apocrine carcinomas outside these conditions is also uncommon, and there are only 32 cases reported in the literature review conducted by Katagiri and Ansai^4^, whose epidemiological aspects are the following: preferential age range over 40 years, absence of gender or race preference, more frequent location in the armpits, but there are reports also on the scalp, chest, frontal region, spleen, hand, finger and lip. This type of tumor has been reported as apocrine carcinoma, apocrine gland carcinoma, duct-papillary apocrine carcinoma, cutaneous apocrine carcinoma or cutaneous ductal apocrine carcinoma^1^, ^3^, ^5^, ^6^, ^7^.

The present study aimed at presenting a case of apocrine carcinoma of the head and neck region, in addition to discussing the aspects related with diagnosis, management and prognosis.

## CASE REPORT

**Figure 1:**
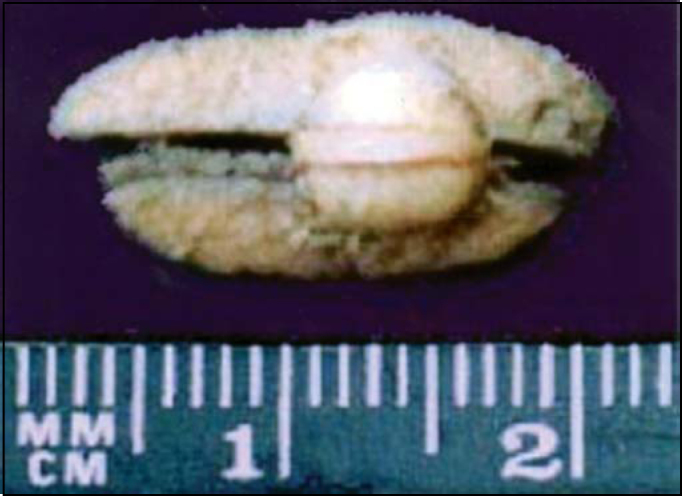
Aspect of cutaneous nodule removed from the dermis of submandibular region (2nd lesion).

Female 51-year-old Black-descendent patient, came to Service of Otorhinolaryngology, University Hospital Antônio Pedro, Universidade Federal Fluminense (HUAP/UFF) reporting performance of exeresis of a cyst (sic) from the left parotid gland in another center. The patient had the removed surgical specimen in fixating substance, which was referred to the Clinical Pathology, where the diagnosis of parotid gland apocrine carcinoma with one impaired surgical margin was made. We conducted thorough clinical examinations and complementary tests to check the presence of primary tumor or metastases. CT scan of the operated region did not show neoplastic or regional lymphoadenopathy affections. We conducted gynecological exams to check the presence of breast and anogenital region neoplasms, complemented by genital cytopathology, breast ultrasound and mammogram. Cutaneous neoplasm was also investigated. We did not find any indicative signs of neoplasm. We recommended proactive observation of the patient. Eight months after, she presented nodular lesion (Figure 1), measuring 0.7 cm diameter, reddish, non-bleeding and pruruginous on the skin of submandibular left region, 1.5cm from the surgical scar. After removal of the lesion, clinical pathology concluded it was cutaneous apocrine carcinoma infiltrated into the deep dermis, with free surgical margins.

**Figure 2:**
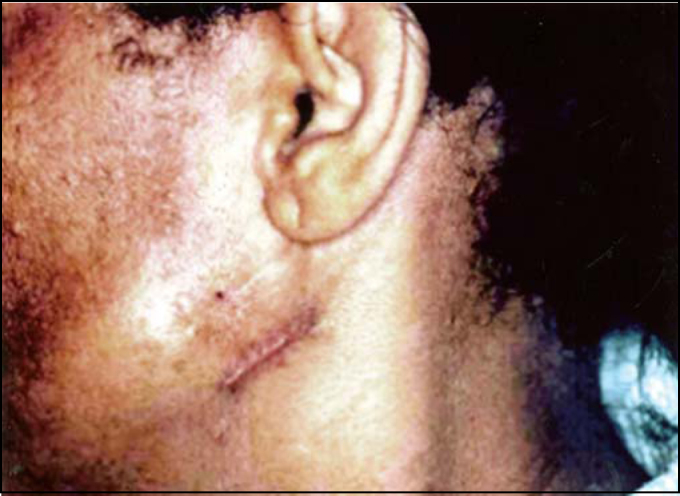
Small tumor in parotid region (3rd lesion). We can also observe the surgical scars of previous lesions removed.

Six weeks later, the patient presented a small tumor on the left parotid region (Figure 2). We conducted surgery to remove the tumor and homolateral lymph nodes. Frozen transoperative biopsy showed that tumor mass was compatible with apocrine carcinoma and surgical margins and regional lymph nodes were free, which was also confirmed by clinical pathology analysis. Patient was indicated for follow up and she did not present any further affections up to one year after the last procedure.

**Figure 3:**
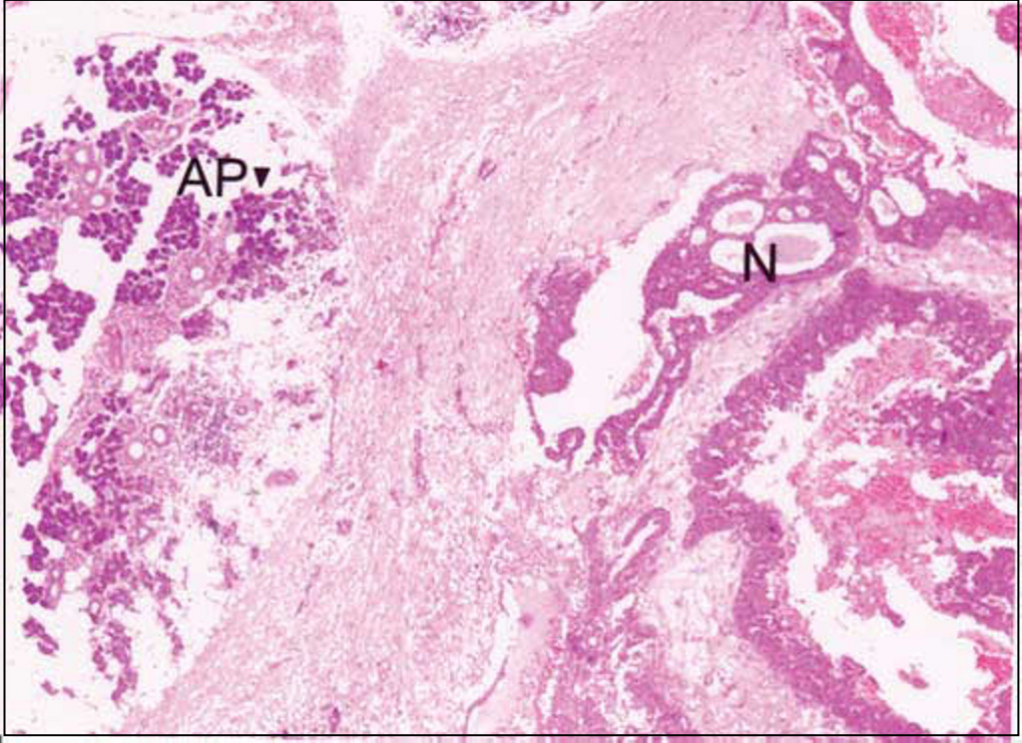
Infiltrative glandular epithelial aspect of neoplasm (N). AP: parotid acines. (HE, objective 4X).

**Figure 4:**
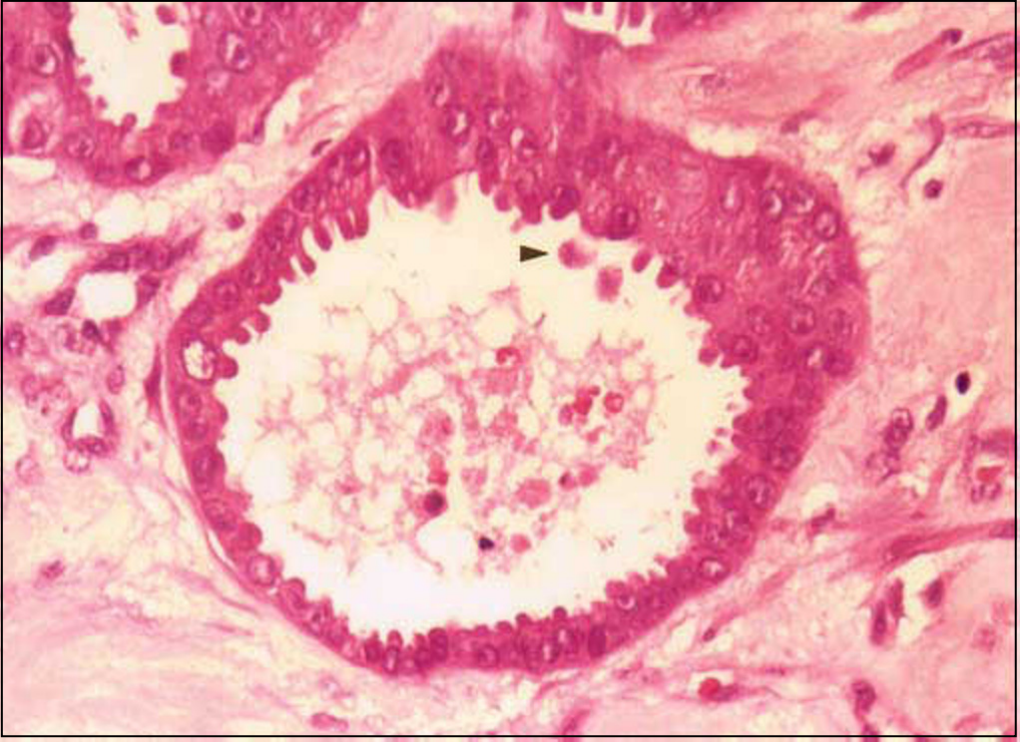
Arrow: secretion by apical decapitation (HE, objective 40X).

Parotid and skin lesions on submandibular region presented the following histopathological aspects of neoplastic tissues: presence of infiltrative glandular epithelial neoplasm (Figure 3), presenting polygonal or rounded cells placed in glandular disposition, of variable size and complexity. These cells had eosinophilic and granular cytoplasm with large vesicular nuclei and prominent nucleoli. There was moderate cell and nuclear polymorphism and mitoses were not frequent. We evidenced the presence of apical decapitation secretion (Figure 4), characteristic of apocrine glands in most tumor cells. We also observed: a) cystic structure of variable diameter containing eosinophilic material; b) tumor cells in glandular lumen forming pads or microcysts; c) sebaceous differentiation in cells with clear cytoplasm randomly distributed or in cohesive aggregates; d) focuses of comedo-necrosis with calcification; and, e) invasion of blood vessels by tumor cells. Stroma presented as dense and fibrous with areas of hemorrhage, hyalinization and foci of dystrophic calcification, in addition to moderate lymphoplasmocytarian infiltrate. Additionally, we found presence of material stained with PAS and no diastase in tumor cells or lumen of neoplastic gland structures.

Final diagnosis of parotid and skin lesions in submandibular region was apocrine carcinoma with cystic adenoid pattern with cribiform areas, based on morphological and histochemical aspects fully compatible with cutaneous apocrine carcinoma and absence of any other primary neoplasm.

## DISCUSSION

To conduct microscopic diagnosis of cutaneous apocrine carcinoma, the presence of apical decapitation secretion in primary skin neoplasm is practically specific for apocrine differentiation^6^.

In addition to the fundamental aspect of apocrine differentiation, well and moderately differentiated apocrine carcinomas are uni or multinodular neoplasms that present glandular conformation and acinar structures that vary in size, disposed side by side or in confluence. Some lesions contain papillary or cystic areas. Margins are typically circumscribed, but normally there is no capsule and it is possible to identify infiltration foci forming cell bands or strings, with or without lumen formation. Neoplastic cells tend to be uniform within a specific tumor; they are cuboid or columnar and characteristically they contain moderate to abundant amounts of cytoplasmatic eosinophilic granules. Nuclear atypia may not be present or be modest in well differentiated apocrine carcinomas, in which there is no frequent mitosis figures. Prominent nucleoli are common^5^.

To complement the information on morphological findings, we can also detect other aspects for the diagnosis of apocrine origin of tumor: presence of iron-positive granules in neoplastic cells^3^, presence of positive PAS material resistant to diastase in tumor cells and lumen, and positive immunelabeling in neoplastic cells^3^; presence of positive PAS material resistant to diastase in tumor cells and in lumen^5^, and positive immunereaction to two or three antigens comprising GCDFP-15 (gross cystic disease fluid protein-15), lisozime and CD-15^4^.

For apocrine differentiated tumor located in the parotid, we can consider the following diagnostic hypotheses for differential diagnosis: 1) metastasis of breast ductal carcinoma owing to the presence of apocrine differentiation that may take place in the tumor, in addition to the fact that the breast is a frequent site of primary tumor in infraclavicular anatomical location, generating metastases to the parotid^7^, ^8^; 2) metastasis of cutaneous apocrine carcinoma that has marked apocrine differentiation^5^, ^6^, ^7^;3) salivary gland ductal carcinoma, whose basic aspect is to have some similarities such as the fact that neoplastic cells present abundant eosinophilic and granular cytoplasm, with increased nuclei and prominent nucleoli, forming micropapillary arrangements or comedo-necrosis foci^9^, ^10^; and 4) renal cancer metastases, in which neoplastic cells also have abundant eosinophilic and granular cytoplasm, which can also present areas of papillary differentiation^7^, ^8^.

In our case, histopathological findings found in parotid and submandibular skin lesions were compatible with extensive cutaneous apocrine carcinoma in analyzed materials. Thus, there is the possibility that the lesion removed from the skin in the submandibular region (second lesion) is the oldest neoplasm, which could have originated a metastasis to the parotid, and after installation, it would have grown quickly and manifested as primary lesion. It is less likely that the parotid tumor was the primary one, in which the metastasis would have got detached and installed in the submandibular skin region, maybe during removal of the first lesion. In turn, the third lesion (of the parotid) should have appeared as recurrence, which is compatible with the histopathological finding of impaired surgical limits in the analysis of the surgical specimen brought by the patient in the first visit.

As to treatment of cutaneous apocrine carcinoma, many authors highlighted that the extensive surgical excision with complete removal of tumor mass is the standard therapy and that it seems to offer the best possibility of cure^2^, ^4^, ^6^, ^11^. Radiotherapy may be used in case of local recurrence or involvement of regional lymph nodes^4^, ^11^. Systemic chemotherapy has not proved to be effective in treating these tumors, even though new studies are still ongoing^11^, ^12^.

Considering the prognosis, skin apocrine carcinoma is normally associated with a non-fatal evolution of the disease, but local recurrences and metastases to regional lymph nodes may occur years after the first excision^3^. Moreover, only moderately or little differentiated lesions produce metastases or lead the patient to death^7^. In 32 cases reviewed by Katagiri and Ansai^4^, there was local recurrence in 11 cases, generation of metastases to lymph nodes in 17 cases and in 4 cases they led the patient to death owing to disease progression.

## CLOSING REMARKS

In the case presented here, we could not certainly define which was the site of the primary tumor, whether the lesion affected the parotid gland, the submandibular skin region, or any other site, given that there was the possibility of having lesions with metastasis of occult primary site. Based on histopathological findings compatible with cutaneous apocrine carcinoma, we considered that the lesion removed from the submandibular skin region was probably the primary neoplasm.
